# Study of the changes in the microstructures and properties of grease using ball milling to simulate a bearing shear zone on grease

**DOI:** 10.1038/s41598-024-60399-7

**Published:** 2024-04-28

**Authors:** Jia Ren, Haopeng Cai, Gaiqing Zhao, Zhuang Xu, Xiaobo Wang

**Affiliations:** 1 State Key Laboratory of Solid Lubrication, Lanzhou Institute of Chemical Physics, Chinese Academy of Sciences, Lanzhou, 730000 Gansu People’s Republic of China; 2 University of Chinese Academy of Sciences, Beijing, 100049 People’s Republic of China; 3 Qingdao Center of Resource Chemistry and New Materials, Qingdao, 266100 Shandong People’s Republic of China; 4 Zibo Innovation Center of High-End Synthetic Lubricating Materials, Zibo, 255000 Shandong People’s Republic of China

**Keywords:** Lithium grease, Shear·degradation, Microstructure, Noise properties, Low-temperature torque, Materials science, Mechanical engineering

## Abstract

The effects of shear degradation on the microstructures and properties of grease were investigated using a planetary ball mill to simulate a bearing shear zone on grease. The microstructure, cone penetration, colloidal stability, rheological properties noise properties, water washout characteristics and low-temperature torque of lithium grease were characterized. The microstructure of the initial lithium grease is a three-dimensional network structure formed by the uniform fibers. The entanglement level is high. As the ball milling shear time increases, the network structure of lithium grease is destroyed and the fibers are sheared to become short. Eventually all of them become short fibers. The performance test of lithium grease reveal that the cone penetration increases, colloidal stability, structural strength, noise properties, water washout characteristics of lithium grease gradually decreased with the increase of ball milling shear time. Additionally, the low-temperature starting torque and running torque of the grease gradually decrease. This phenomenon occurs due to changes in the microstructure of lithium grease. The shear degradation of lithium grease was mainly divided into two stages: the rapid stage was the destruction of the thickener network structure and the fibers being shortened by shearing. The slow stage was the process in which short fibers were sheared into shorter fibers.

## Introduction

Rolling bearings are key components of the modern mechanical industry, and they are widely used in machine tools, wind power systems, automobiles and aviation equipment^1–6^. Grease is the fifth component of rolling bearings and plays the roles of reducing friction and preventing wear of rolling bearings. Grease is important to guarantee the long life and stable operation of rolling bearings^7,8^. Grease is mainly composed of base oil, thickener and additives. The thickener has a three-dimensional network structure, and the base oil is a semifluid material^9,10^. The degradation of the grease performance during service is one of the key factors contributing to bearing failure. The friction stress of bearing components in the operation of bearing components is the main factor leading to grease degradation. Grease degradation is mainly divided into shear degradation, thermal degradation and oxidative degradation. Shear degradation is one of the main grease degradation methods. Shear degradation can have a significant effect on the physicochemical properties, flow characteristics and bearing operating performance of grease^11,12^. Therefore, improvement of the grease performance depends on a full understanding of the changes in the grease performance under shear conditions. This understanding of grease ensures its rational design and improvement of the performance optimization efficiency.

It is very difficult to study grease aging due to shear by directly taking samples from bearings due to the complexity of the operating factors of grease in bearings during service. The development of a simulation test machine that can accurately control the shear aging factors is an important solution^13^. Rezasoltani et al. investigated the correlation between mechanical degradation and entropy generation of grease based on irreversible thermodynamic theory. The correlation was verified using a rheometer, and the results showed that mechanical degradation and entropy generation exhibit a linear relationship^14^. Yuxin et al. set up a Couette aging machine with controlled rotational speed (shear rate) and time. The effect of shear on the rheological properties of lithium grease at room temperature was investigated using this test rig. The mechanism of shear aging was hypothesized based on an analysis of the microscopic morphology of the thickener^13^. Khonsari et al. obtained greases with different aging periods using a grease shear tester. The contact angles of the aged samples were evaluated. A correlation between the contact angle and the degree of degradation of mechanically aged samples was established^15^. Leif Ahme et al. investigated the changes in the rheological properties of greases with different thickeners during shear using a rheometer. The effect of shear on the grease properties was analyzed from the point of view of mechanical energy. The degradation of lithium grease and calcium grease thickeners requires less mechanical energy consumption than that of polyurea greases^16^.

To investigate the performance changes and degradation mechanisms of grease during shear degradation, experimental methods that can simulate the shear of grease in bearings must be explored. The shear of grease in bearings is mainly caused by the friction stresses of the bearing rolling element, cages, and inner and outer rings. The friction types of these components are mainly rolling friction and sliding friction^17^. However, the present research methods cannot simulate the shear that grease is subjected to in bearings. Planetary ball mills utilize the relative motion of balls and milling vials to shear a material. The friction types of these balls and vials mainly include rolling friction and sliding friction, which are very similar to the shear and friction types of bearing components^18,19^. It is a good idea to study the changes in the microstructures and properties of grease during shear degradation using planetary ball mills to simulate a bearing shear zone on grease. In addition, the existing research on shear degradation of greases was mainly carried out in terms of energy changes or changes in basic physicochemical properties. However, a study of the effect of shear degradation on the operating performance of grease in bearing rigs and its change mechanism has not been reported.

In this paper, the effects of shear degradation on the microstructures and properties of grease were investigated using a planetary ball mill to simulate a bearing shear zone on grease. First, the lithium grease samples were shear aged for different periods using a ball mill. Then, the microstructures of the lithium grease samples were characterized. The cone penetration, colloidal stability, rheological properties, noise properties, water washout characteristics and low-temperature torque of the lithium grease samples were tested and analyzed. The influence mechanisms of shear on the microstructure and properties of grease were investigated.

## Experimental procedures

### Materials

500N base oil was purchased from Golden Zen Co., Ltd. Lithium 12-hydroxy stearate was provided by Qingdao Lubemater Lubrication Materials Technology Co., Ltd. Heptane was obtained from Shanghai Aladdin Biochemical Technology Co., Ltd. The physical and chemical properties of 500N base oil are shown in Table 1.

**Table 1:** Physical and chemical properties of base oils.

Property	500N	Test method
Kinematic viscosity (40 °C)/mm^2^·s^−1^	91.20	ASTM D 445
Kinematic viscosity (100 °C)/mm^2^ s^−1^	10.48	ASTM D 445
Viscosity index (VI)	97	ASTM D 2270
Pour point/°C	− 19	ASTM D 5950
Flash point/°C	248	ASTM D 92
Density (15 °C)/kg/L	0.87	ASTM D 4052

### Preparation of lithium grease

Lithium grease was prepared by referring to the methods in the present literature^20,21^. The greases were prepared using a multifunctional 5 L kettle with stirring, heating and cooling accessories. The preparation process was as follows. In the kettle, 1880 g of 500N base oil was added. Stirring and heating were initiated. Then, 420 g of lithium 12-hydroxy stearate was added to the kettle, and the temperature was increased to approximately 100 °C. The temperature was maintained and stirred for 60 min. Then, the temperature was slowly increased to approximately 210 °C, and the temperature was maintained and refined for 10 min. Then, 1200 g of 500N base oil was added quickly. The mixture in the reactor was cooled to room temperature. The mixture was first ground three times with a precision three-roller mill, then filtered and degassed to obtain a sample of lithium grease, labeled Li.

The prepared lithium grease (Li) was divided into different batches and processed by planetary ball milling for 4 h, 8 h, 12 h and 16 h. The prepared lithium grease added for each experiment was 200 g. The processed samples were labeled Li-4, Li-8, Li-12 and Li-16. The balls (mean roughness: 30 nm, HRC hardness: 62–65) and milling vials were made of 52,100 standard steel. The diameters of the balls were 15 mm, 10 mm and 5 mm. the total number of balls is 300, 100 of each type. The ball milling rotational speed was 800 ± 10 r/min, and the temperature was about 25 °C. The planetary ball mill was produced by Beijing Guohuangaoke Co., Ltd.

### Characterization of lithium grease

The thermal properties of the lithium 12-hydroxy stearate samples were tested by differential scanning calorimetry (DSC; 204HP, Netzsch). The test temperature was increased from room temperature to 240 °C in a nitrogen atmosphere with a ramp rate of 10 °C/min. The microstructure of lithium grease was characterized using a scanning electron microscope (SEM; JSM-7610F, JEOL). The lithium grease sample had to be pretreated before testing. The pretreatment method was as follows: the lithium grease sample was coated with a very thin film on top of a copper mesh. Then, the sample was carefully put into a reagent bottle containing heptane with tweezers and immersed for 24 h. Afterward, the sample was replaced with heptane solvent and immersed again, and the immersion was repeated three times. The sample was carefully removed with tweezers, dried, and observed after spraying gold.

### Physical and chemical property tests

The cone penetration of the lithium grease samples was tested according to international organization for standardization (ISO) 2137. The dropping point test was performed according to ISO 6299. Colloidal stability characterization was performed by the conical sieve method, and the test method was performed according to American society for testing and materials standard (ASTM D) 6184.

### Rheological property test

The rheological properties were tested using a rheometer (MCR 302, Aton paar). The storage modulus, loss modulus and the recoverability of viscosity after instantaneous high shear were tested. The tests were performed using a plate–plate attachment. The spacing between the test plates was 1 mm. The storage modulus and loss modulus were tested for shear strains ranging from 0.01 to 150%. The test temperatures were 40 °C and 80 °C. The intersection of the energy storage modulus and loss modulus curves was the flow point where the grease transforms into a fluid. The shear stress at the flow point represented the structural strength of the lithium grease. The viscosity recoverability properties were tested at 40 °C and 80 °C with instantaneous maximum shear rates of 3000 s^−1^ and 2000s^−1^, respectively. The percentage of the viscosity of the lithium grease at 60 s after the instantaneous high shear rate to the viscosity at the beginning of the test was calculated from the test data. The viscosity recovery rate of lithium grease after an instance of instantaneous high shear represents the structural recovery ability.

### Noise property test

The noise properties were tested using a grease noise test rig (Bequiet + , SKF). The test bearings were 608 bearings. The tested rotational speed was 1800 rpm, the axial load was 30 N, and the temperature was 25 °C. The test method was based on the Bequiet + standard test procedures. Each sample was tested twice to ensure reproducibility. The noise classes of the greases were categorized as GNX, GN0, GN1, GN2, GN3 and GN4. The GN classes start with the worst class of GNX and end with the best class of GN4. The noise class of the greases was determined based on the percentage of the BQ class. The BQ class was determined based on the vibration peak value of greased 608 bearings. The BQ class was divided into four levels with maximum bearing vibration peaks of 5 μm/s, 10 μm/s, 20 μm/s, and 40 μm/s.

### Water washout characteristics test

The water washout characteristics test of grease utilized a grease water resistance test rig (BFZ-48, North Dalian). The test was carried out with reference to standard ISO 1109. A typical 6204 bearing was used for the test bearing, and the test temperature was 38 °C.

### Low-temperature torque test

A low-temperature torque test was performed with a low-temperature grease torque test rig (Model 64–21, Lawler). The test method for grease was based on the national petrochemical industry standard SH/T 0338. The test was performed at − 30 °C.

## Results and discussion

### DSC of lithium 12-hydroxy stearate

**Figure 1:**
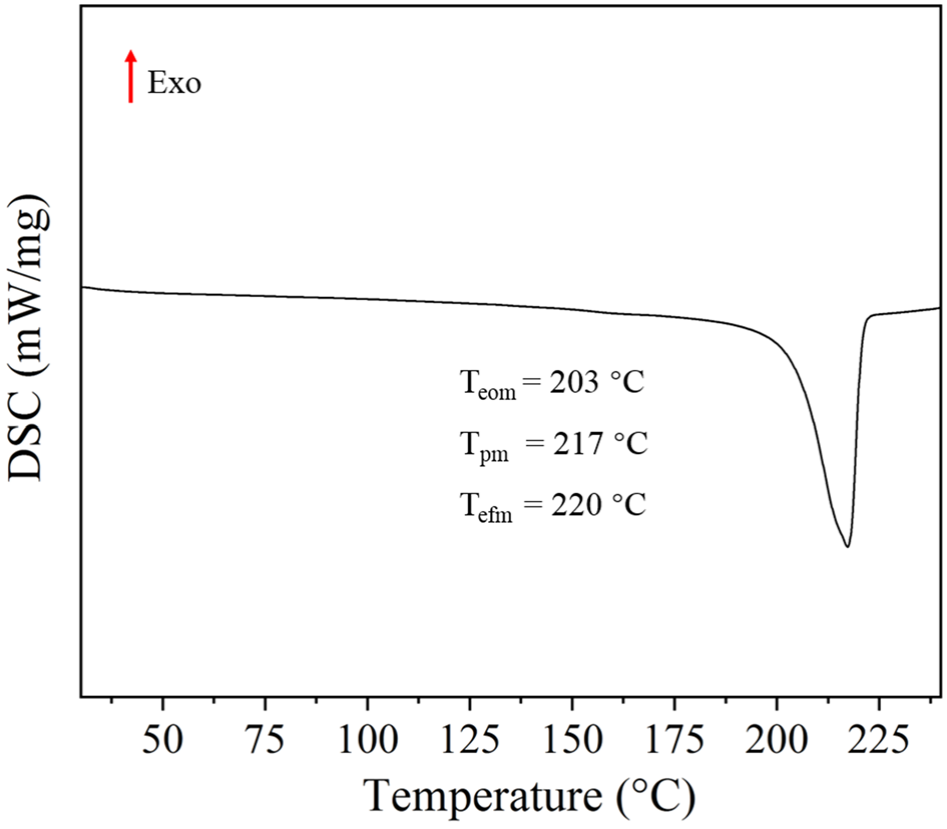
DSC curve of lithium 12-hydroxy stearate.

DSC is commonly used to study the melting behavior of materials. Figure 1 shows the DSC curve of lithium 12-hydroxy stearate. Lithium 12-hydroxy stearate has an extrapolated onset temperature (T_eom_), a melting peak temperature (T_pm_) and an extrapolated final temperature (T_efm_) of 203 °C, 217 °C and 220 °C, respectively.

### Microstructure of lithium grease

**Figure 2:**
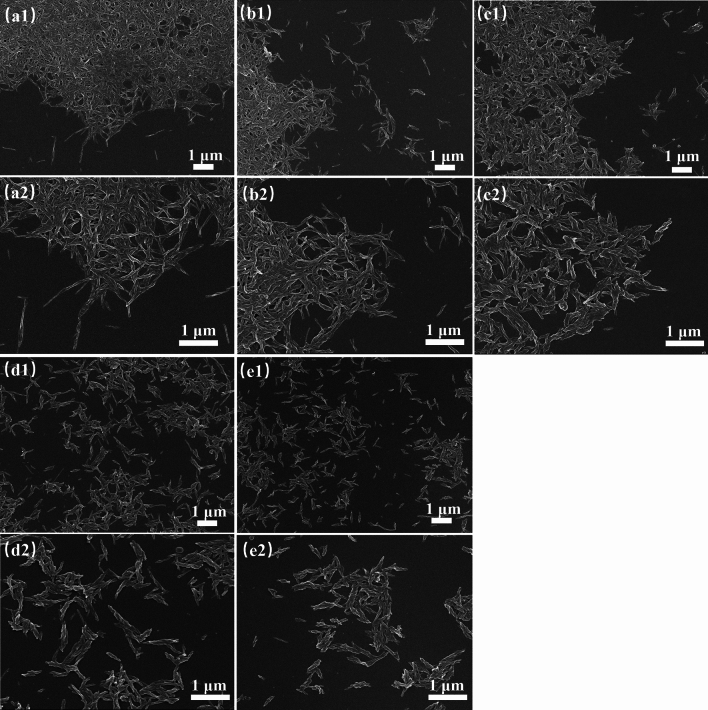
SEM images of lithium greases: (**a1**,**a2**) Li, (**b1**,**b2**) Li-4, (**c1**,**c2**) Li-8, (**d1**,**d2**) Li-12, and (**e1**,**e2**) Li-16.

Figure 2 shows SEM images of different lithium greases. The thickener of Li has a network structure formed by uniform fibers. The degree of fiber entanglement is high, as seen in Fig. 2a1,a2. With increasing ball milling shear time of lithium grease, the thickener fiber length gradually shortens, and the degree of entanglement decreases. Compared with the Li thickener, a few short fibers appear in the Li-4 thickener, indicating that the network structure is mildly destroyed. After 8 h of grinding, the fibers of the Li-8 thickener are relatively loose, the degree of entanglement is reduced, and the number of short fibers is increased, as shown in Fig. 2c1,c2. When the ball milling shear time of lithium grease is further increased to 12 h, the thickener is composed of almost all short fibers. This result indicates that the network structure is severely damaged and that a large number of fibers are fractured, as shown in Fig. 2d1,d2. The fiber structure of Li-16 is similar to that of Li-12, but the fiber length is shorter than that of Li-12. This result occurs due to the destructive effects of shear on the thickener network structure and fibers produced by the planetary ball mill simulation.

### Physical and chemical properties of lithium grease

**Figure 3:**
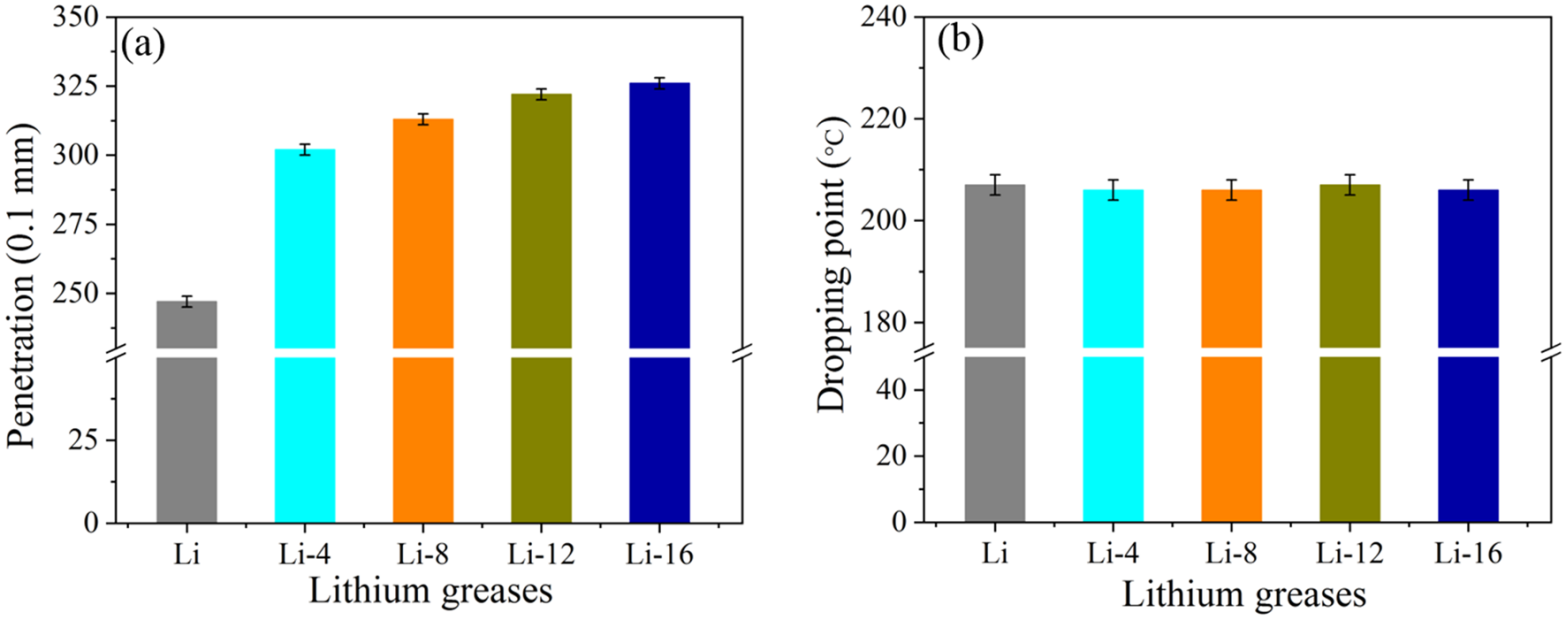
Penetration (**a**) and dropping points (**b**) of lithium greases.

Figure 3 shows the cone penetration (a) and dropping points (b) of the lithium grease samples. As shown in Fig. 3a, the cone penetration of the lithium grease gradually increases with increasing ball milling shear time. The cone penetration of Li-4, Li-8, Li-12 and Li-16 increases by 22.27%, 26.72%, 30.36% and 31.98%, respectively, relative to Li. The structure of the initial lithium grease thickener is a three-dimensional network composed of fibers. First, the shear during ball milling destroys the connecting points of the three-dimensional network structure of the thickener. Moreover, the thickener fibers become shorter under shear. This leads to a significant increase in grease cone penetration. When the milling time is further increased, the shear has a small destructive effect on the short fibers, and the process of the short fibers being sheared into shorter fibers is slow. The change in grease cone penetration is not significant^13^. As shown in Fig. 3b, there is no significant change in the dropping point of lithium grease with increasing milling time. Shear thinning has no significant effect on the dropping point of lithium grease. This phenomenon occurs because the dropping point of lithium grease is mainly determined by the melting point of the lithium 12-hydroxy stearate thickener (Fig. 1), and the structure of the thickener has no significant effect on its dropping point.

**Figure 4:**
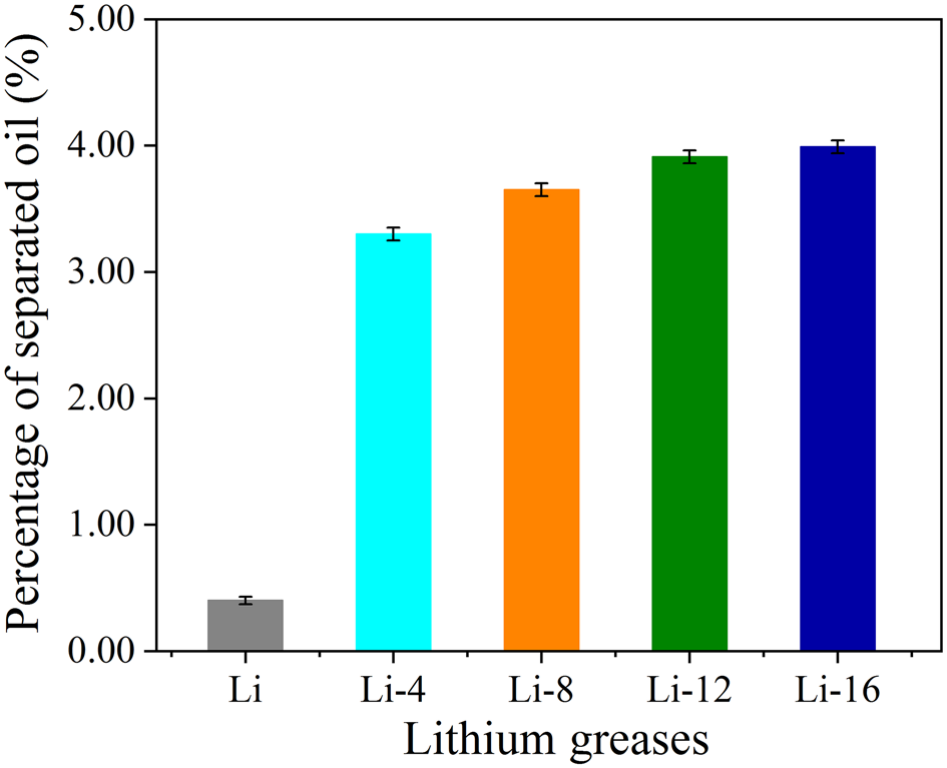
Oil separation properties of lithium greases.

The colloidal stability of grease is generally characterized by the mass percentage of separated base oil; the conical sieve method is commonly used for this characterization. Figure 4 shows the oil separation percentages of the lithium grease samples. Compared with the conical sieve oil percentage of Li, with increasing ball milling shear time, the percentage of oil separation of lithium grease gradually increases, and the colloidal stability gradually deteriorates. This phenomenon occurs due to the shear damage to the network structure and the fiber structure of the lithium grease thickener, and the binding effect on the base oil is reduced, resulting in an increase in the percentage of separated base oil.

### Rheological properties of lithium grease

**Figure 5:**
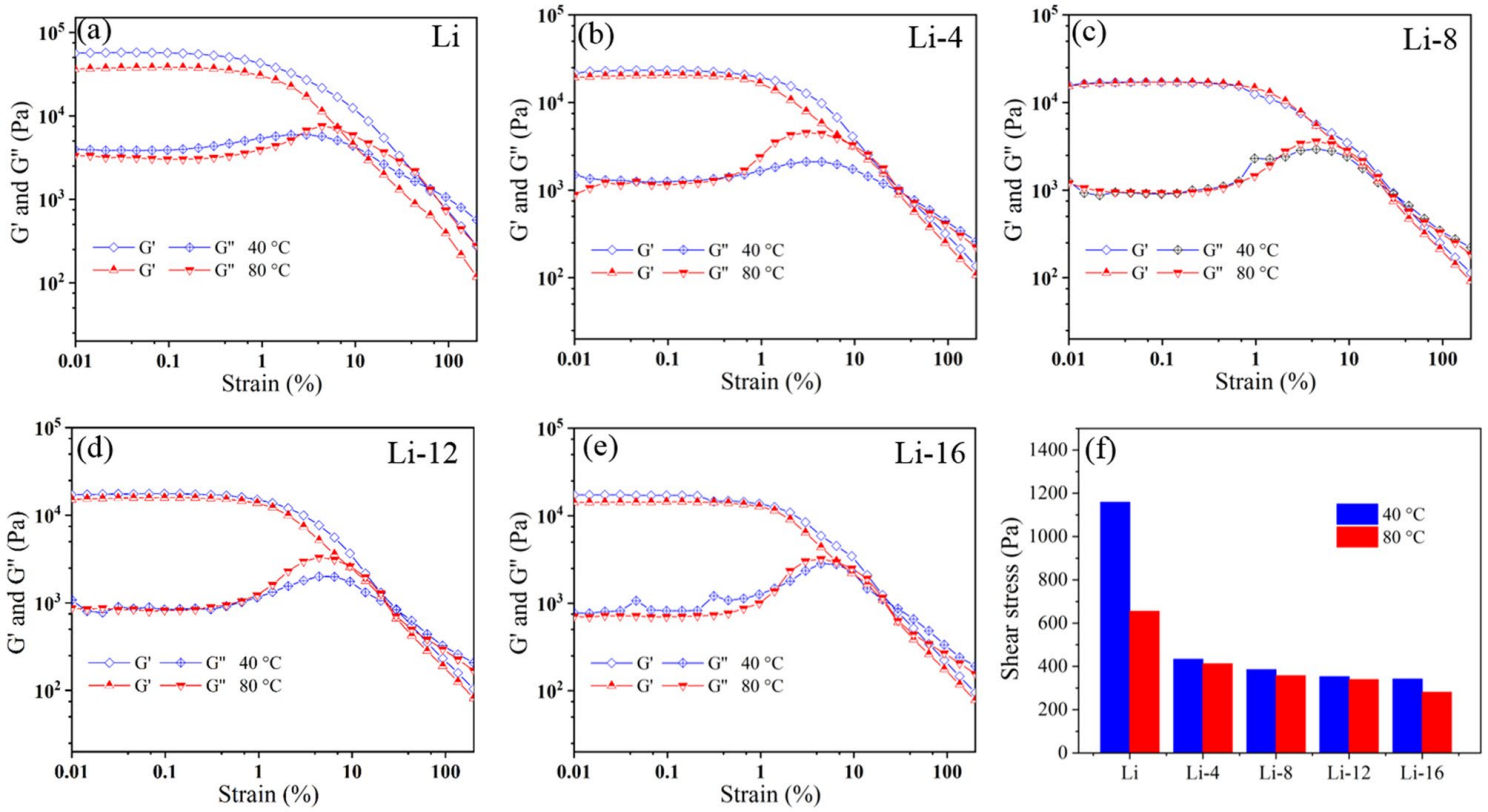
Rheological properties of lithium greases: (**a**) Li, (**b**) Li-4, (**c**) Li-8, (**d**) Li-12, and (**e**) Li-16 storage modulus (*G′*) and loss modulus (*G″*) curves. (**f**) Flow point shear stresses of lithium greases.

Figure 5 shows the storage modulus and loss modulus curves of the lithium grease samples at 40 °C and 80 °C and a histogram of the flow point shear stress. The flow point shear stress represents the structural strength of the grease. A high shear stress at the flow point corresponds to a high structural strength of the grease. Conversely, the grease has a low structural strength for a low shear stress. Figure 5a–e show that all the lithium grease samples exhibit viscoelasticity. The storage modulus and loss modulus curves enter a nonlinear viscoelastic region after going through a linear viscoelastic region, and they intersect at a point. The lithium grease becomes fluid after the intersection point^22,23^. Before 12 h, the storage modulus and loss modulus of the lithium grease samples gradually decrease with increasing ball milling shear time. After that, the decreases in the storage modulus and loss modulus are not significant. Compared with the storage modulus and loss modulus of the lithium grease samples at 40 °C, only Li shows significant decreases in the storage modulus and loss modulus at 80 °C. The other lithium grease samples show no significant decreases in their storage modulus and loss modulus. As shown in Fig. 5f, the flow point shear stress of Li is high, and the structural strength is high. Compared with the flow point shear stress of Li, the flow point shear stresses of Li-4, Li-8, Li-12 and Li-16 are reduced by 62.64%, 66.78%, 69.54% and 70.49%, respectively, at 40 °C and by 37.09%, 45.49%, 48.24% and 57.09%, respectively, at 80 °C. The decrease in the flow point shear stress occurs mainly before 12 h, which is consistent with the structural changes in lithium grease. In the early stages of ball milling shear, the three-dimensional network structure of the thickener and the fibers are rapidly destroyed under shear. The thickener becomes dispersed short fibers. The storage modulus, loss modulus and flow point shear stress are significantly reduced. When the ball milling time is further increased, the process of changing the short fibers into shorter fibers under shear becomes slower. The storage modulus, loss modulus, and flow point shear stress decrease by smaller degrees than before^24^. When the temperature increases, the thermal motion of the thickener molecules increases, and the molecular spacing increases. The connecting points of the three-dimensional network structure and the force between the thickener molecules decrease; thus, the structural strength of Li significantly decreases. For the milled lithium grease samples, the three-dimensional network structure is destroyed, and the structural strength change is affected by temperature to a lesser degree^25^.

**Figure 6:**
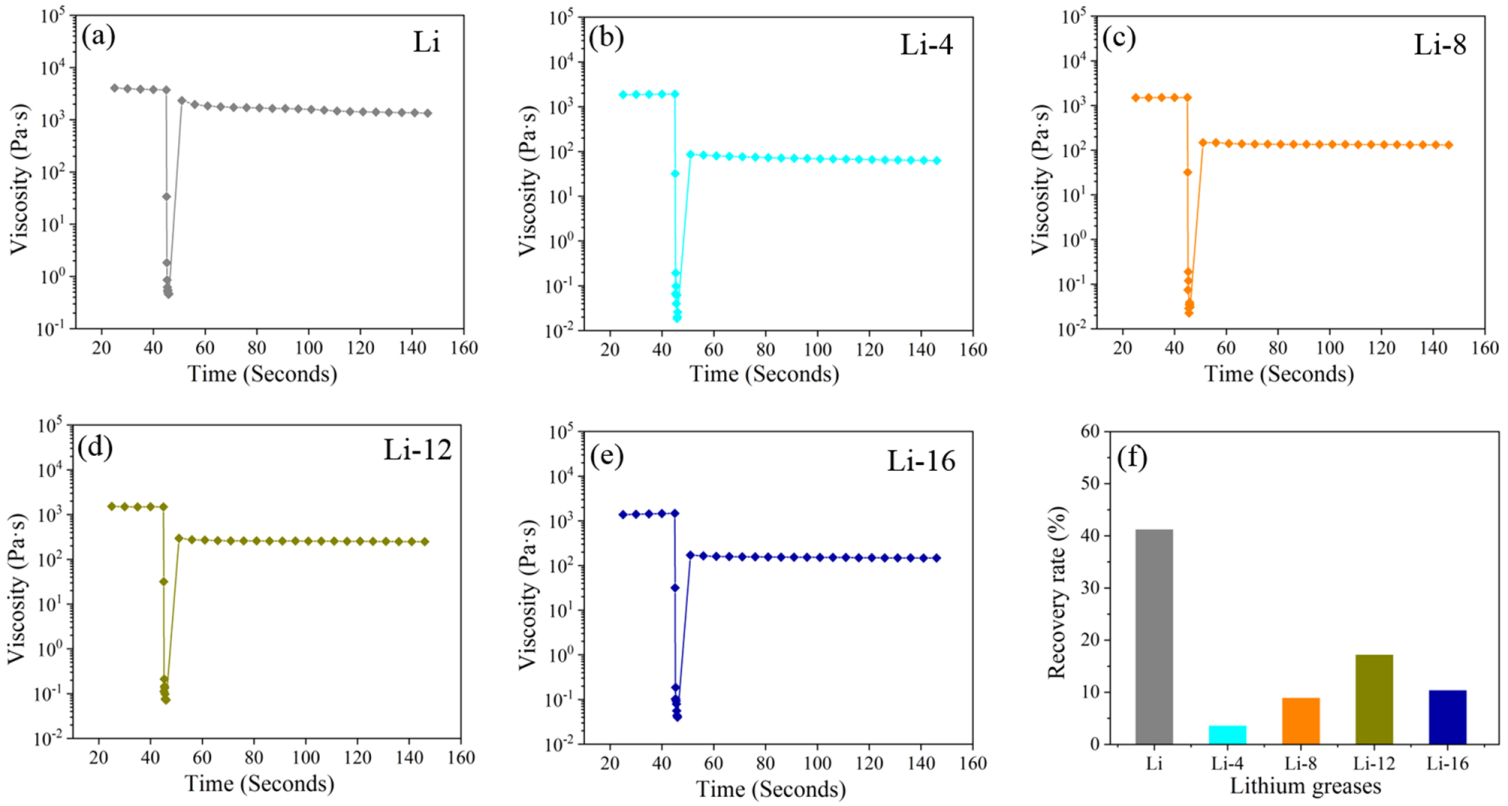
Thixotropic curves of different lithium grease samples under instantaneous shear (maximum shear rate of 3000 s^−1^) at 40 °C: (**a**) Li, (**b**) Li-4, (**c**) Li-8, (**d**) Li-12, and (**e**) Li-16. (**f**) Recovery rate of different lithium grease samples.

**Figure 7:**
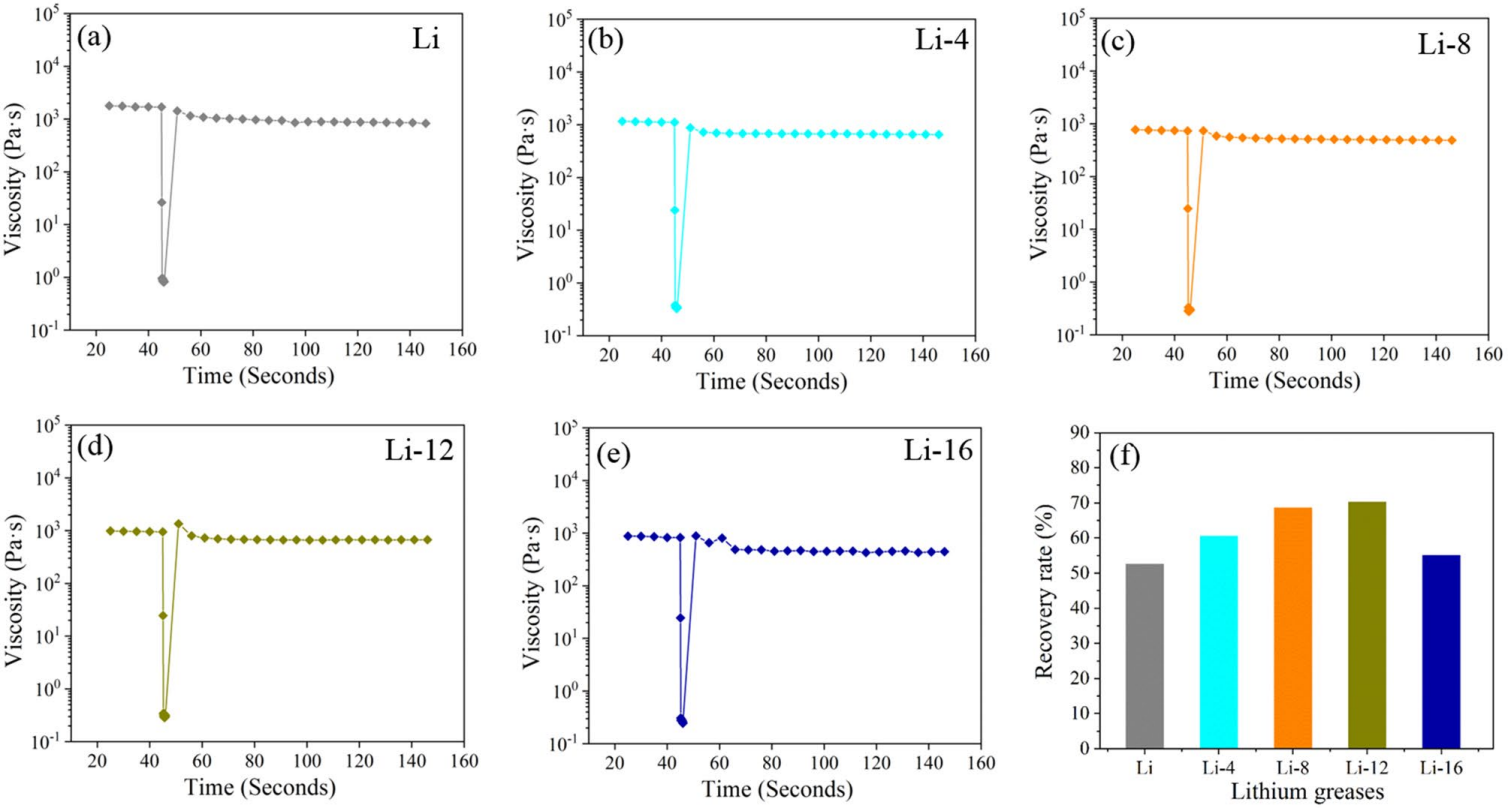
Thixotropic curves of different lithium grease samples under instantaneous shear (maximum shear rate of 2000 s^−1^) at 80 °C: (**a**) Li, (**b**) Li-4, (**c**) Li-8, (**d**) Li-12, and (**e**) Li-16. (**f**) Recovery rate of different lithium grease samples.

The viscosity change curves and viscosity recoverability levels of the lithium grease samples at 40 °C and 80 °C are shown in Figs. 6 and 7. The viscosities of the lithium grease samples are relatively high at the low shear rate at the beginning of the test (before 40 s). The viscosities sharply decrease at the instantaneous high shear rate and finally stabilize at a relatively low state after partial recovery at a low shear rate. At 40 °C, the viscosity of lithium grease significantly decreases after the instantaneous high shear rate, which is due to the destruction of the structure of lithium grease under instantaneous high shear. Lithium grease (Li) has the highest viscosity recoverability, and the viscosity recoverability levels of the milled samples are reduced. This phenomenon occurs because the structure of the Li thickener is three-dimensional and formed by fibers, which can be connected and quickly recover after instantaneous high shear. The three-dimensional structure of the ball milled samples has been severely damaged; it is further damaged after undergoing the instantaneous high shear of the rheometer, and structure recovery is difficult. When the temperature is 80 °C, the viscosity of the lithium grease samples decreases by less after undergoing instantaneous high shear. This phenomenon occurs because the initial viscosity of the lithium grease decreases with increasing temperature, so at high temperatures, the initial viscosity is low, and the viscosity minimally decreases after undergoing instantaneous high-speed shear, which is manifested as a high viscosity recovery rate^26^.

### Noise properties of lithium grease

**Figure 8:**
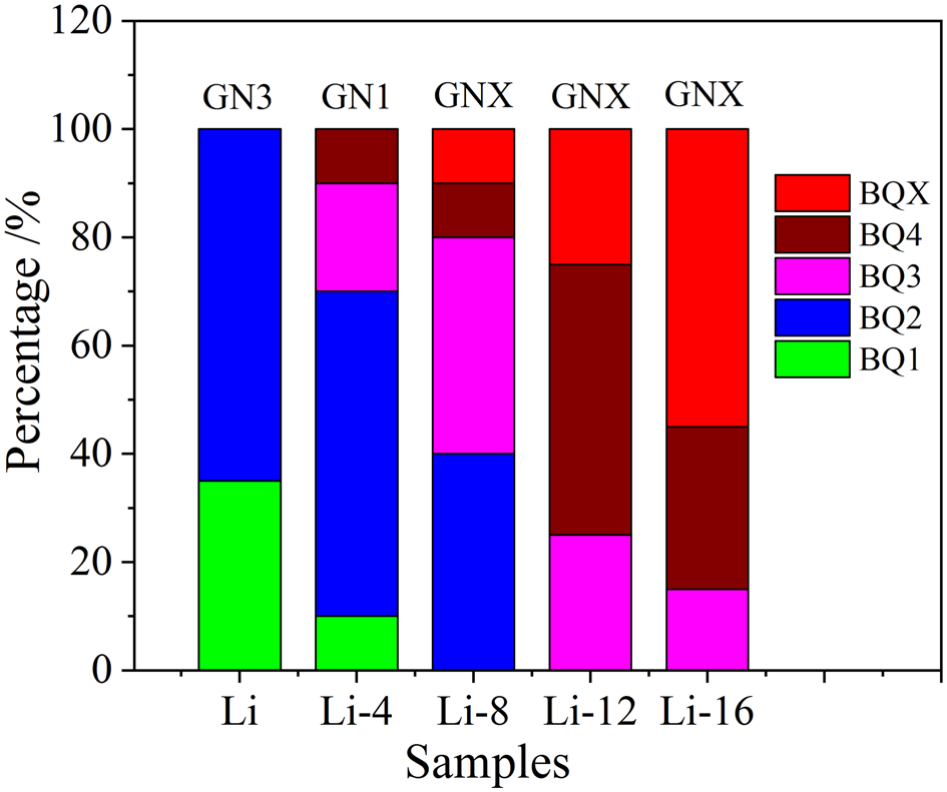
Noise classes of lithium greases.

Noise is an important property of precision bearing greases. Bearing noise is mainly caused by component friction, wear and abnormal collisions. Grease is an important lubricating material to reduce bearing noise. The effects of ball milling shear on the noise properties of the lithium grease samples were further investigated using the Bequiet + rig. The noise performance of the lithium grease samples is shown in Fig. 8. The noise class of Li is GN3, which is the best class among all the samples. The noise class of Li-4 is GN1, and the noise classes of Li-8, Li-12 and Li-16 are all GNX. Comparing the percentage of BQX for samples Li-8, Li-12 and Li-16, there is a gradual increasing trend. The noise class of lithium grease decreases with increasing milling time. Shear degradation leads to a reduction in the noise performance of lithium grease. The thickener of the initial lithium grease has a network structure formed by the connection of long fibers. This microstructure has a relatively high strength and a strong adsorption level, which are conducive to the formation of a thick lubricating protective film. Therefore, bearing noise can be well reduced. After ball milling shear, the lithium grease thickener network structure and fibers are destroyed, the fibers are shortened and loosened, the microstructure strength is reduced, and the formation of the lubricating protective film is weakened. Consequently, the performance levels of bearing noise reduction are poor^27^.

### Water washout characteristics of lithium grease

**Figure 9:**
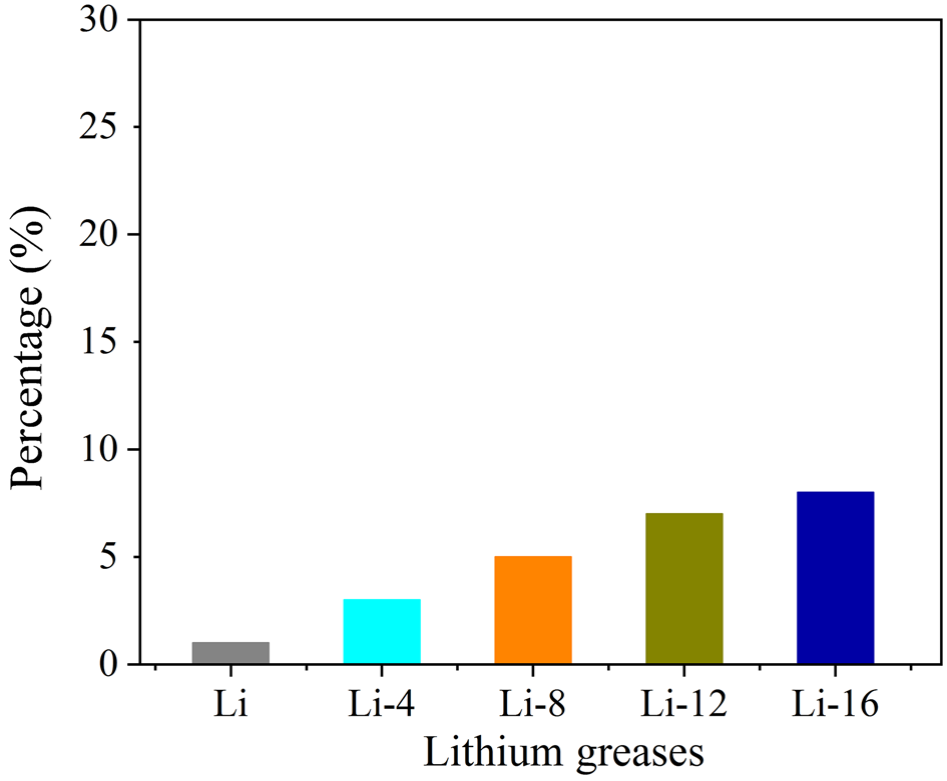
Water washout characteristics of lithium greases.

The water washout characteristics indicate the ability of grease to resist being flushed out of the bearing by water. The percentage of mass of grease washed away by water represents the ability of the grease to resist water. A smaller percentage indicates that the grease has good resistance to water washout. The water washout resistance of the lithium greases is shown in Fig. 9. The lithium grease sample Li shows the best resistance to water flushing. When the lithium grease is sheared by ball milling for longer periods of time, the grease becomes less resistant to water washout. The thickener with a three-dimensional network structure has a high structural strength, so it has good resistance to water washout. The thickener consisting of dispersed short fibers has a low structural strength, resulting in poor resistance to water washout.

### Low-temperature torque of lithium grease

**Figure 10:**
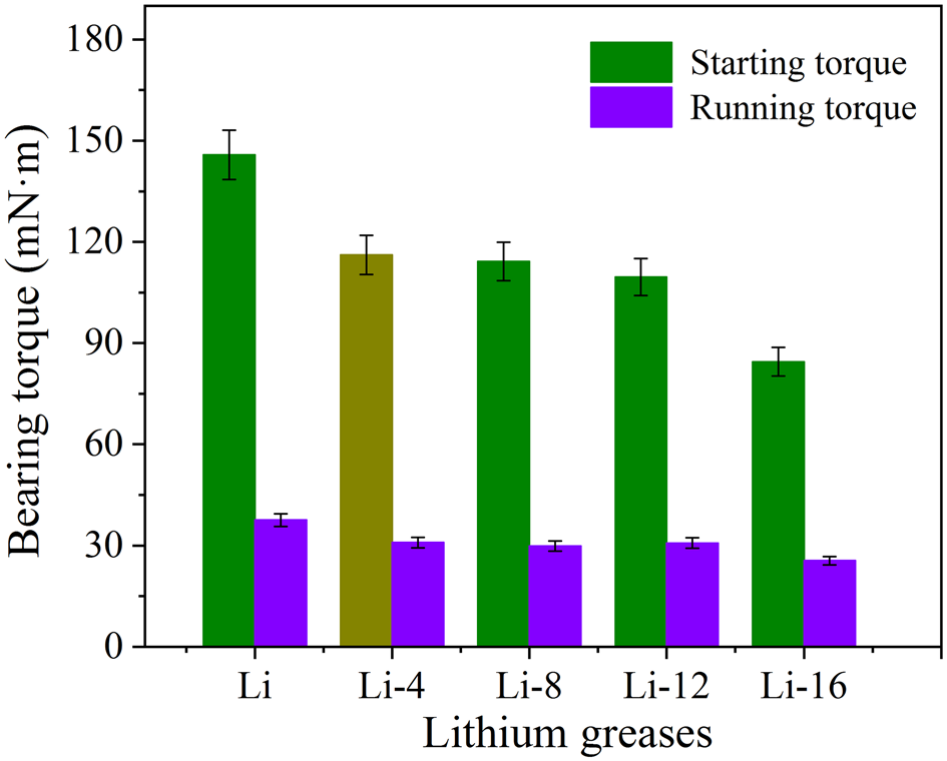
Low-temperature starting torque and running torque of lithium greases at − 30 °C.

The low-temperature starting torque and running torque represent the resistance of grease to bearing operation at low temperatures. Low-temperature starting torque and running torque are important parameters for evaluating the low temperature performance of grease. Figure 10 shows the low-temperature starting torque and running torque of lithium greases at − 30 °C. It is clear from the graph that the ball milling shear leads to a decrease in the starting torque and running torque of the grease. The low-temperature starting torque is reduced by 20.32%, 21.65%, 24.85% and 42.05% for Li-4, Li-8, Li-12, and Li-16, respectively, and the low-temperature running torque is reduced by 17.70%, 20.47%, 18.13% and 32.09% for Li-4, Li-8, Li-12, and Li-16, respectively, compared to the low-temperature starting torque and running torque of Li. The three-dimensional network thickener structure with a high structural strength has a high running resistance to bearings at low temperatures, so the low-temperature starting and running torques are high. The short fiber structure with a low structural strength and a low degree of entanglement has a low running resistance to bearings at low temperatures, so the starting and running torques at low temperatures are low^28^.

### Degradation mechanism analysis

**Figure 11:**
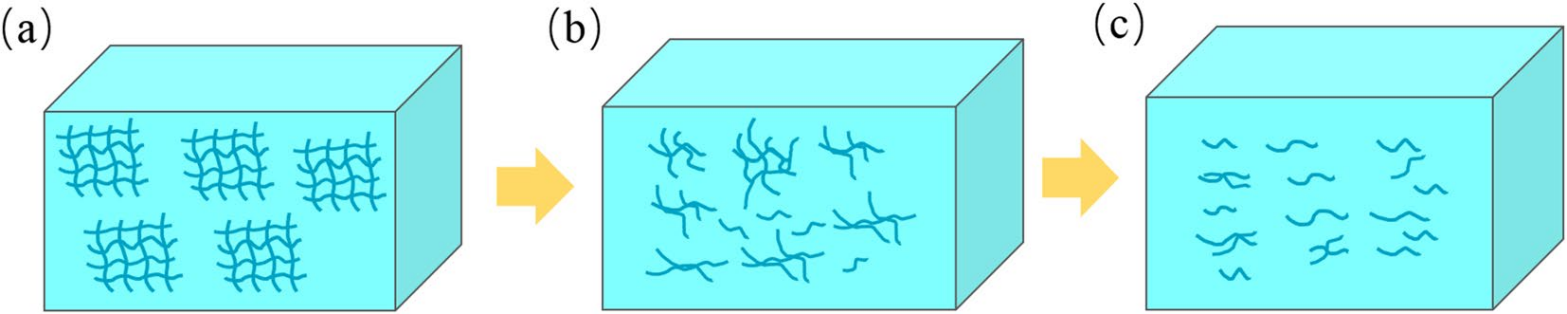
Schematic diagram of the degradation mechanism.

Shear destroys the grease thickener network structure and fibers, leading to shear degradation of the grease and changes in the grease performance. This affects the service performance of the bearing^29^. The shear degradation of the lithium grease thickener is mainly divided into two stages. The first stage is the destruction of the thickener network structure and the fibers being shortened by shearing. At this stage, the connection points of the network structure composed of fibers are broken, and some of the fibers are sheared into short fibers. The thickener develops a structure with a very low degree of entanglement and a large number of short fibers (Fig. 11b)^13^. The second stage is the process in which short fibers are sheared into shorter fibers. The fibers of the thickener are almost all changed into shorter fibers (Fig. 11c). This process is relatively slow. The microstructure of the thickener is the determining factor for the lithium grease performance. Generally, shear degradation of the thickener leads to deterioration of the grease performance. The cone penetration of lithium grease significantly increases in the early stage of ball milling shear (before 12 h), which is attributed to the first stage of shear degradation. In the later stage of ball milling shear (after 12 h), the increase in cone penetration is slow, which is mainly attributed to the second stage of shear degradation. The colloidal stability and structural strength of lithium grease exhibit similar trends. The initial lithium grease (Li) with a three-dimensional network structure has a better viscosity recoverability performance after instantaneous high shear at 40 °C, whereas the viscosity recoverability performance after instantaneous high shear is poor for the ball-milled grease. Degradation of the thickener structure of lithium grease hinders the noise properties and water washout characteristics. The reason for this phenomenon is mainly the change in the microstructure of the thickener leading to a reduction in the structural strength of the grease. However, the degradation of grease under shear has a positive effect on the starting and running torques of bearings at low temperatures. The short fiber structure of grease after shearing by ball milling has low starting and running torques at low temperatures.

## Conclusions

The use of ball milling to simulate the shear of a bearing on grease was realized. The three-dimensional network structure and fibers of lithium grease were destroyed, leading to changes in the lithium grease performance. With increasing ball milling shear time, the microstructure of lithium grease changed, resulting in a gradual increase in the cone penetration, whereas the colloidal stability, structural strength, noise properties and water washout characteristics gradually decreased. Additionally, the low-temperature starting torque and running torque of the grease gradually decrease. The performance changes of lithium grease were relatively fast in the early stage of ball milling shear. In the later stage of ball milling, the performance changes of lithium grease were slow. The shear degradation of the microstructure of lithium grease was mainly divided into two stages: the rapid stage was the destruction of the thickener network structure and the fibers being shortened by shearing. The slow stage was the process in which short fibers were sheared into shorter fibers. This result was consistent with changes in grease properties.

## Data Availability

The data used in this research is available from the corresponding author on reasonable request.
